# Clinical Observations on Diseases of the Rectum

**Published:** 1866-01

**Authors:** James R. Lane

**Affiliations:** Surgeon to St. Mary’s and Lock Hospitals, and to St. Mark’s Hospital for Diseases of the Rectum


### CLINICAL OBSERVATIONS ON DISEASES OF THE RECTUM.

By JAMES R. LANE, F.R.C.S.,
Surgeon to St. Mary's and Lock Hospitals, and to St. Mark's Hospital for Diseases of the Rectum.

### ON THE ALLEGED DANGERS OF THE LIGATURE IN THE OPERATION FOR HÆMORRHOIDS; AND THE COMPARATIVE MERITS OF THE TREATMENT BY THE CLAMP AND BY THE LIGATURE.

There is perhaps no operation in the whole range of surgery which affords so much relief at so small a cost of suffering and risk as the removal of hæmorrhoical tumors by the well-known and long-established operation with the ligature. Nevertheless there appears still to be, on the part of the public, and even of many members of the medical profession, a sort of undefined dread of ill consequences arising from it; the result of which is, that many persons are induced to linger on for years in misery and discomfort, when surgery is capable of affording them complete and permanent relief, at a risk scarcely greater than they are constantly incurring in the ordinary affairs of life.

A good illustration of what I state has been recently afforded me in the case of a young officer, who had spent some years in India, and who was suffering from the disease in its most aggravated form. It completely incompacitated him for his duties, and compelled him to return to this country for treatment. When I saw him he had been here more than a year, but had nothing done, because he had been told by a physician of eminence, whom he consulted, that it would cost him his life if he allowed himself to be meddled with. He was otherwise in fair health, and could discover nothing in his case to contraindicate an operation. I therefore persuad[illegible] him to submit, and the consequence was that he recovered [illegible]hout any untoward symptom, and was quite well at the end of a fortnight.

I have no doubt that to the existence of this vague impression might be traced the introduction of nitric acid in the treatment of this disease; and now, since experience has shown this remedy to be quite inadequate to the cure of the complaint in its severer forms, and only applicable to a small and exceptional class of cases, I believe we owe to a similar cause the introduction of a new operation with a clamp and the actual cautery. This method has been persistently vaunted during the last two or three years, as affording a certain means of cure combined with a complete immunity from all those indefinite ills to which the ligature has been stated to give rise. I have no wish to depreciate this new operation, which is ingenious enough, and may, I doubt not, be found effectual, though I do not believe it to be better, and there is not a shadow of proof that it is safer, than the methods previously in use. I cannot, however, refrain from expressing my opinion that its author, Mr. H. Smith, has exceeded the limits even of parental partiality, in the way in which he has attempted to build up its reputation at the expense of the fair fame of our old and tried friend, the ligature.

The operation to which I allude is performed in this wise. The hæmorrhoidal tumors being protruded, they are seized and their basis firmly compressed with a clamp. They are then cut off, and, to prevent bleeding, the cut surface is cauterized, either with nitric acid or the hot iron, the latter having been found the more effectual. The clamp is then removed, and the cut surface, covered of course by the layer of slough produced by the cautery, is allowed to recede into its place within the rectum. Any external piles which may happen to be present are then cut away with the knife or scissors.

Shortly after the introduction of this plan of operation, we were told by Mr. Smith, in some clinical remarks, that, "admirable as was the method of treating these diseases by the ligature, it was not free from danger to life; and if the surgeon possessed some means equally effectual, and yet free from dangerous consequences, he is bound to employ them; and therefore he was glad to be able to recommend, in the strongest manner, the improved clamp," &c. Shortly afterwards, I read that by this method "the dangers of the ligature are entirely avoided." And more recently I have met with the following statement:— "It is impossible for any surgeon conscientiously to tell his patient that there is no danger whatever after the ligature; but this may be *most truthfully stated* with regard to the operation by my improved clamp; if the most ordinary precautions are taken to prevent bleeding." Again: It is *not possible* that either tetanus or pyæmia, the two most formidable results of the ligature, can occur after this operation, because the condition which produces the former affection does not obtain—viz., the presence of and irritating substance around the nerves for several days; and pyæmia, or other inflammatory affections, will be effectually prevented by the exposed surface being deprived of its vitality, and the veins being blocked up by the cauterization." The pathological views enunciated in the paragraph last quoted are of so novel and startling a character that they impel one to ask whether tetanus has hitherto been unheard of after burns, and how long the application of the actual cautery to cut surfaces has been known to be an effectual guarantee against pæymia and "other inflammatory affections." In the interests of science, and for the credit of English surgery, I cannot help entering my protest against such reckless assertions as these, unsubstantiated as they are by a shadow of logical proof, and without even the slender recommendation of *a priori* probability to sustain them.

But what is the *real* state of the case as regards the consequences of the operation with the ligature, deducible from the experience of those who have had the largest opportunities of forming a judgment on such a matter? The following quotation from the writings of Sir B. Brodie must commend itself to everyone by its plain common sense and sound judgment. He says:—"I conceive this is not only one of the most effectual, but one of the safest operations in surgery. I should think I must have performed or seen it performed between 200 and 300 times. I saw one patient who died after the operation, in consequence of diffuse inflammation of the cellular membrane running up the inside of the gut as high as the mesentery, but that was a patient whose constitution was broken down by long-continued hæmorrhage and in whom any slight accident might have produced equally bad consequences. I saw another patient who a week after the operation, and having been quite well in the interval, had an attack of pain in the abdomen and shivering, attended with fever and died------With the exception of these two cases, I never knew any ill consequences arise. I contend therefore that the operation is as safe as any operation can be expected to be. You are not to suppose that even the slightest operations in surgery are absolutely in all cases free from every particle of danger, any more than the slightest accident. I have known two patients die after the extraction of a tooth, and I have known several die in consequence of venesection of the arm, or an accidental prick of the finger. The chance of danger from this operation is at any rate so trifling that you need not calculate upon it. If you were to calculate upon so small a chance as this, you would scarcely be able to do anything in the ordinary affairs of life." Again, what is the opinion of the leading surgeon of the northern capital, who is well known to have had had for many years a most extensive practical experience in such cases. Mr. Syme says: "I feel warranted, after very extensive employment of the ligature, to state that it may be used without the slightest risk of any serious inconvenience. Indeed, in the whole course of my practice I never met with a case which terminated fatally, or threatened to do so, when the ligature simply was employed."

My own experience of the operation with the ligature amounts now to as many as 427 cases. In the immense majority the progress towards recovery has been singularly uniform. It has been rare indeed that I have met with any untoward symptoms, or that the healing has been much delayed; and I have never seen any cause for serious alarm, except in two cases which occurred more than seven years ago, and which have already been made public. The two cases to which I allude proved fatal from tetanus. They were lodged in the same ward, and were seized with tetanus on the same day, at a time when that complaint was epidemic in the hospitals of London to a remarkable, and I believe almost an unprecedented, extent. Out of the whole number of 427 cases which I have treated, I have never seen an instance of pæymia, or erysipelas, or of diffuse inflammation. Mr. Gowlland, my colleague at St. Mark's Hospital, must have operated on as nearly as possible the same number, his appointment to that institution having been made at the same time as my own, and I know that his experience is almost identical with mine on this question. He has never met with a case of pyæmia, and has had no fatal result, excepting, strange to say two cases of tetanus, which occurred within a short time of those to which I have already referred, and were fairly attributable to the influence of the same epidemic.

Tetanus and pyæmia, as we have seen are alleged to be "the two most formidable results of the operation with the ligature." With respect to tetanus, there is no doubt it will occasionally follow this, as it will also occasionally follow every conceivable surgical proceeding. By careful search in the medical journals it would not be difficult to find cases in support of such an allegation against almost every operation in surgery, whether small or great. But I contend that there is no evidence that the ligature of hæmorrhoids has any special liability to produce this disease, and that there is no ground whatever for the statement that the inclusion of tissues in a ligature is a more probable exciting cause than the cauterization of the same tissues with the hot iron. In point of fact, burns have long been rather unfavorably known for their tendency to be followed by tetanus; therefore, in contrasting the cautery with the ligature, the inference to be drawn is certainly not to the disadvantage of the latter. The assertion that it is *not possible* for tetanus to follow the operation with the clamp and cautery is too absurd to require refutation, and equally so is the argument that an operation is exempt from an exceptional complication like this because twenty, fifty, or even a hundred cases have not furnished an example of it.

Again, with respect to pyæmia, it is to be feared that this is an occurrence from which no surgical operation will ever be able to claim complete exemption, especially in large hospitals. But all our knowledge on the subject tends to show that it depends on the health and condition of the individual patient, and on his atmosphere and other surroundings, rather than on the nature of the operation. It will be time enough to argue against the singular theory, that a slough produced by cautery are materially different in their predisposing influence, when any reliable facts are adduced in its support.

But I am in a position to assert confidently, and on positive grounds, that the operation with the ligature does *not* possess any peculiar liability to pyæmic infection. On the contrary, the reverse according to my experience has been remarkably the case, so much so that I have long been surprised at the exemption. I have the authentic testimony of 427 cases occurring in my own practice, and reliable information of about an equal number in the practice, of my colleague, thus making a total of about 850, and out of this large number not a single instance of pyæmia has been observed.

Having now shown, I trust conclusively, that there is no ground, either in reason or fact, for the assertions which have been made in favor of the clamp at the expense of the ligature, as regards immediate danger to life, I would ask to be permitted to examine some of the other grounds on which it has been pressed into public notice. We are told by the author of the operation "that it is not only on account of its far greater safety that he practises and strongly recommends the employment of his clamp in cases of internal hæmorrhoids and prolapse, but he adopts it for the additional and important reason, that the period of convalescence after this operation is rendered so brief that the patients are enabled to be up and about their business *in one-third of the time* which is of necessity consumed after the ordinary operation with the ligature." The majority of the patients are stated to have been "moving about" in four or five days, though in some it is admitted that the convalescence was protracted over a period of a fortnight or three weeks.

I am unable to speak from direct personal experience of the comparative merits of the two operations. I have not yet employed the clamp, in consequence of a scepticism, for which I trust I shall be pardoned, with regard to its alleged extraordinary merits, and also because it appears to me to possess the disadvantage of being more painful and protracted at the time, and of being, in severe cases, by no means certainly free from a liability to hæmorrhage subsequently. This liability, indeed, is evident from Mr. Smith's account of his own cases. I have, however, had the advantage of repeatedly seeing it performed by my colleague, Mr. Gowlland, who has used it in, I believe, about twenty cases. Having witnessed most of that gentleman's operations, I can testify to the careful and complete manner in which every detail was carried out. I have seen most of these cases after the operation, I have made particular inquiry respecting their progress, and I have found that there has not been, as a rule, less pain than after the other operation, nor has the convalescence been in any degree accelerated. They have not been considered fit to be discharged from the hospital in a less time than the average of other cases. My colleague, Mr. Allingham, informs me that he has operated with the clamp and cautery on fourteen cases, and he has arrived at precisely similar conclusions. I, therefore, take the liberty of demurring to the statement that the recovery will be complete in one-third of the time, and that patients will, as a rule, be able to go about their business in four or five days.

But I must also demur to that statement for the following additional reasons:—In the great majority of cases requiring operation there is more or less redundancy of the outer skin, "external piles," requiring removal. These must be cut away with the knife or scissors, whichever operation is adopted; and the sores resulting from their removal will render the majority of patients extremely averse, from "moving about" more than they can possibly help, at the end of four or five days. But where the outer skin is not inflamed or redundant, and does not require to be interfered with, but the disease is altogether internal, and the parts admit of being resorted to their natural position with the sphincter, as soon as the operation is concluded,—in such cases, I contend, the patient will be quite as well able to "move about" after the one operation as after the other. Whether it is prudent to allow him to move about is another question. I am strongly of opinion that it is not; indeed in private practice I not unfrequently find it necessary to repress the tendency to do so, because I believe that where there are large sloughing sores in the interior of the rectum (whether produced by ligature or cautery it cannot matter one straw) it is of the first importance that rest and the recumbent position should be observed, at all events until healthy granulation has commenced. If this salutary rule is largely departed from, I venture to predict that untoward results will soon become more frequent, whether the operation be performed by the clamp or by the ligature.

But it has been also alleged as an argument against the ligature and in favor of the clamp, that sometimes "the wounds resulting from the separation of the ligatures would not heal up for a long period, and the patient would be subjected to much painful suffering, necessitating, perhaps, some other operation." According to my experience, cases of this kind are very uncommon, though I admit, of course, that they may occur; indeed, after any operation, out of a large number of cases, delay and obstinacy in the healing process must always be occasionally met with; but I would ask, on what conceivable surgical principle should such a result not be equally likely to follow the operation with the clamp and cautery. Wounds made with the cautery, on the outside of the body at all events, have never been noted for any peculiar rapidity in cicaterization.

I repeat that I have no desire to depreciate the operation with the clamp. I believe it to be a sufficiently effectual method of removing the disease, but I consider it to be in many respects inferior, and in no respect to have the advantage over, the operation with the ligature. It is, in truth, only another mode but a less certain and complete mode, of doing the same thing. I admit, however, that it is an immense step in advance over the treatment by nitric acid, with which the name of its author was some years since so much identified. I should never have thought of saying a word on the subject had the new operation been left to stand upon its own merits, and had not comparison, which I consider to be unjustifiable and unfair, been made between the two operations. I should be delighted to welcome any *real* improvement in the treatment of this disease, but I cannot admit that this is likely to be effected by the clamp. On the other hand, I consider that the operation with the ligature has done incalculable service to humanity, and my belief is that it will continue to do so long after the clamp, like many other ingenious novelties, has been laid aside and forgotten.

In conclusion, I would add a few words respecting the best mode of operating with the ligature. The plan which I invariably adopt is that invented by Mr. Salmon, which is, in my opinion, far superior to any other. By means of it most of the objections which can fairly be urged against the ligature are obviated. The hemorrhoidal tumors being protruded, they are seized in succession with a hook or vulsellum, and are separated with the scissors from the subjacent parts, along the line of union between skin and mucous membrane. The cut made with the scissors should be sufficiently deep and free to detach the tumor from the muscular tissue on which it rests to an extent equal to about three-fourths of its base, leaving it attached by its upper fourth only. The ligature is placed in the deep groove thus made, and is tied tightly round the upper attached part. The danger of hemorrhage is securely provided against, because the vessels which supply the tumor do not enter it indiscriminately at its base, but descend from above beneath the mucous membrane, and their trunks are therefore necessarily included in the ligature. When the ligatures have been tied, the bulk of the tumors may be cut away, care being taken to leave sufficient tissue beyond the ligature to prevent it from slipping, and the parts may then be returned within the sphincter. Any redundancy of external skin which is apparent *after the parts have been returned* must be cut off with the scissors. This plan of operation is far preferable to the usual method of strangulating the whole base of the tumor by transfixing it with a needle, and tying it in two halves—first, because it is completed in less than half the time; secondly, because scarcely more than one-fourth the amount of tissue is strangulated by the ligature—indeed the ligature need not include much more than the trunks of the vessels supplying the tumor; thirdly, because the ligature is tied round mucous membrane at some distance from the anus, where the sensibility is much less acute, and consequently the after pain and irritation are very greatly diminished. Instead of the whole attached base of the tumor being converted into a sloughing wound, as when the whole of it is included in the ligature (and equally, I might add, when the whole of it is burnt with the hot iron,) three-fourths of it is made into a simple incised wound with the scissors, and the sloughing process is confined to the upper fourth only.—*London Lancet.*

